# Increase of cytosolic phospholipase A2 as hydrolytic enzyme of phospholipids and autism cognitive, social and sensory dysfunction severity

**DOI:** 10.1186/s12944-016-0391-4

**Published:** 2017-06-15

**Authors:** Hanan Qasem, Laila Al-Ayadhi, Hussain Al Dera, Afaf El-Ansary

**Affiliations:** 1 Biochemistry Department, Science College, King Saud University, P.O. Box 22452, 11495 Riyadh, Saudi Arabia; 2 Autism Research and Treatment Center, Riyadh, Saudi Arabia; 3 Shaik AL-Amodi Autism Research Chair, King Saud University, Riyadh, Saudi Arabia; 4 Department of Physiology, Faculty of Medicine, King Saud University, Riyadh, Saudi Arabia; 5 Basic medical science dept. College of Medicine, King Saud bin Abdul Aziz University for Health Sciences, Riyadh, Saudi Arabia; 6 King Abdullah International Medical Research Center (Kaimrc), Riyadh, Saudi Arabia; 7 Central Laboratory, Center for Female Scientific and Medical Colleges at King Saud University, Riyadh, Saudi Arabia

**Keywords:** Autism, Phospholipids, Cytosolic phospholipase A2, Neuroinflammation, Oxidative strss, Short sensory profile, Childhood autism rating scale, Social responsiveness scale

## Abstract

**Background:**

Autism is neurodevelopmental disorder that is characterized by developmental, behavioral, social and sensory abnormalities. Researchers have focused in last years in immunological alteration and inflammation as a hot subject in autism field. This work aims to study the alteration in phospholipids (PE, PS, and PC) together with the change in cPLA2 concentration as the main phospholipid hydrolytic enzyme in autistic patients compared to control. It was also extended to find a correlation between these biomarkers and severity of autism measured as childhood autism rating scale (CARS), Social responsiveness scale (SRS), and Short sensory profile (SSP).

**Methods:**

Phospholipids (PE, PS, PC) and cPLA2 as biochemical parameters were determined in the plasma of 48 Saudi autistic male patients, categorized as mild-moderate and severe as indicated by their Childhood Autism Rating Scale (CARS), social responsiveness scale (SRS) and short sensory profile (SSP) and compared to 40 age- and gender-matched control samples.

**Results:**

The reported data demonstrate significantly lower levels of PE, PS, and PC together with a significant increase in cPLA2. While association between severity of autism and impaired phospholipid concentration was completely lacked, an association between cPLA2 and impaired sensory processing was observed.

**Conclusions:**

The impaired phospholipid level and remarkable increased in cPLA2 concentration asserted their roles in the etiology of autism. Receiver operating characteristic analysis together with predictiveness diagrams proved that the measured parameters could be used as predictive biomarkers of clinical symptoms and provide significant guidance for future therapeutic strategy to re-establish physiological homeostasis.

## Background

Autism is a complex neurodevelopmental disorder that manifests before three years and considered as one of the most widespread disorder of childhood with high rate of morbidity, impact on the family and cost to society. According to recent epidemiological data, around one in fifty children is affected with autism [1]. The disorder is characterized by developmental, behavioral, social and sensory abnormalities [2, 3]. There is a significant gender bias in autism, with approximately 4:1 male/female ratio [4]. Its cause remains unknown, but it considered as a multi-factorial disorder that is influenced by genetic, immunological, and environmental factors with oxidative stress as a mechanism linking these risk factors [5].

Individual with autism exhibited cognitive disability, memory reduction and they have self-focused attention [6]. In general, cognitive level was associated with autism severity [7]. It’s not surprising that all autistic children have unusual response to auditory, visual and tactile stimuli [8, 9]. There are a few studies that examined sensory profile in autistic children. Children with autism had more sensory dysfunction (i.e., tactile sensitivity, auditory, taste and smell) compared to children with other developmental delays when they measured by SSP [10, 11].

Neuroinflammation are characterized by brain cell activation (i.e., microglia and astrocyte) and increase in cytokine production as major causes of cell damage in autistic children [12, 13]. Evidence suggest that mast cells activation participate to a modulation of blood brain barrier (BBB) [14, 15]. Biochemical studies have shown elevated inflammatory cytokines such as IL-1, IL-6, IL8, IL12, and tumor necrosis factor (TNF-α) in serum, plasma, and cerebral spinal fluid in autistics [13, 16, 17]. It has been proposed that abnormal activation of neuroinflammation considered as potential mechanism in autism pathogenesis.

Several studies have been concluded the impairment in glutathione (GSH) associated pathway in autism [18–20]. These finding have shown that brain cells induce and release diverse inflammatory mediators in response to oxidative stress [21–23]. Phospholipase A2 and cyclooxygenase-2 are target proteins for inflammation and usually induced by proinflammatory factors such as cytokines, infections and peroxidants [24–26].

Neuronal membranes are rich in lipids, and can contain up to 80% lipid by weight [27]. These membranes are composed of glycerophospholipids [phosphatidylcholine (PC), phosphatidylethanolamine (PE), phosphatidylserine (PS) and phosphatidylinositol (PI)], sphingolipid and cholesterol [28]. The cellular membrane plays a protective, anti-inflammatory role and indirectly an antioxidant role, favoring physiological defense processes against free radicals. In addition to their role as structural components of the cell membrane, phospholipids serve as precursors for various second messengers such as arachidonic acid (AA), docosahexaenoic acid (DHA), ceramide, 1,2-diacylglycerol, phosphatidic acid, and lysophosphatidic acid [29]. Neuronal membrane phospholipids are more susceptible to reactive oxygen species ROS because they are rich in polyunsaturated fatty acids (PUFAs) that are labile to peroxidation and oxidative damage [30]. In addition, it is not particularly enriched in antioxidant defenses [20, 31].

Reduced level of polyunsaturated fatty acids have been associated with autism [32–35]. Autism also implies to be associated with changed in lipid metabolism which can be participating in the pathogenesis of this disease. These changes aggravate alterations in the cell membrane phospholipid function and structure. A significant reduction in phospholipid levels in the plasma of autistic patient have been previously identified [33, 36].

In addiation, children with autism have been shown higher phospholipase A2 (PLA2) activity compared to their matched control [32]. Evidence proposes that the instability observed in fatty acid levels may be caused by an increase in PLA2 activity, perhaps in association with the high oxidative stress found in autistic patients [37]. Recent studies have been proved that deregulation of lipid metabolism due to PLA2 over activation is associated with nervous system dysfunction and cognitive impairment [34, 35, 38].

Childhood Autism Rating Scale (CARS), Social Responsiveness Scale (SRS) and Short Sensory Profile (SSP) have been used as scales to define children with autism and determined the severity and abnormalities of autistic behaviors beside build strong background about their social and psychological problem [39–41]. In this context, the aim of this study was to evaluate the relationship between impaired plasma phospholipid levels, cPLA2 activity and the age, cognitive, social and sensory profiles of children with autism compared with healthy control subjects.

## Methods

### Participants

The study protocol followed the ethical guidelines of medicine Collage, King Saud University according to the most recent Declaration of Helsinki (Edinburgh, 2000). All subjects enrolled in the study (48 autistic children and 40 control males) had filled informed consent and signed by their parents. They were enrolled through the ART Center (Autism Research & Treatment Center) clinic in King Khalid University Hospital in Riyadh. The ART Center clinic population consisted of children diagnosed on the autism spectrum disorder (ASD). The diagnosis of ASD was confirmed in all subjects using the Autism Diagnostic Interview- Revised (ADI-R) and the Autism Diagnostic Observation Schedule (ADOS) and Developmental, dimensional diagnostic interview (3DI). The mean of age of all autistic children participated in the study were between 7 ± 4 years old. All were simplex cases. All were negative for fragile x gene study. The control group recruited from pediatric clinic at King Saud medical city in Riyadh with mean age 7 ± 4 years old. Subjects were excluded from the investigation if they had dysmorphic features, or diagnosis of fragile X or other serious neurological (e.g., seizures), psychiatric (e.g., bipolar disorder) or known medical conditions. All participants were screened via parental interview for current and past physical illness. Children with known endocrine, cardiovascular, pulmonary, liver, kidney or other medical disease were excluded from the study.

### Behavioral assessment

The CARS score was fulfilled as a scale for autism severity. CARS assess the child on a scale from one to four in each of 15 dimensions or symptoms (relating to people; emotional response; imitation; body use; object use; listening response; fear or nervousness; verbal communication; non-verbal communication; activity level; level and reliability of intellectual response; adaptation to change; visual response; taste, smell and touch response; and general impressions). Total Scores at or above 30 strongly suggest the presence of autism. Children who have scored 30–36 have mild to moderate autism (*n* = 23), while those with scores ranging between 37 and 60 points have severe autism (*n* = 27) [39]. SRS is the first widely used quantitative parent/teacher-report measure of autistic behaviors; it was completed in 15 to 20 min. A total score of 76 or higher is considered severe and strongly associated with a clinical diagnosis of autistic disorder. A score of 60–75 is interpreted as falling in the mild to moderate range of social impairment [40].

**Fig. 1:**
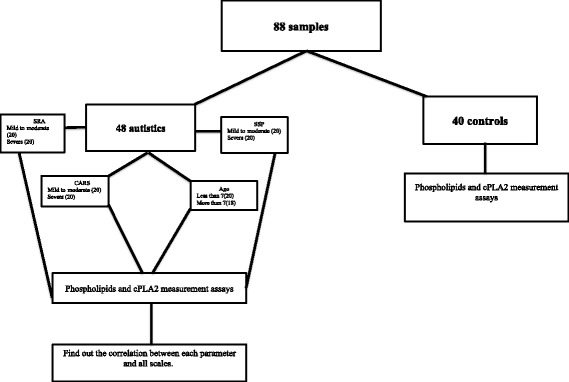
Illustration of the studied groups, demonstrating control group and autistic patients with mild-moderate and severe SRS, CARS, and SSP

The Short Sensory Profile (SSP; Dunn 2001) is a 38-item questionnaire intended to rates a variety of sensory impairments. Each item on the SSP is measured on a 5-point Likert scale. Domain scores are measured in the areas of tactile sensitivity, taste/smell sensitivity, movement sensitivity, seeking sensation, auditory filtering, low energy levels, and visual/auditory sensitivity. Domain scores, as well as overall sensory response, are categorized as typical performance, probable difference from typical performance, and definite difference from typical performance. The score less than 142 consider as severe (definite difference), score from 142 to 152 consider as mild to moderate (probable difference) and score from 153 to 190 consider a typical performance. SSP can provide information about the sensory processing skills of children with autism to assist occupational therapists in assessing and planning intervention for these children [41]. The studied control and autistic groups are illustrated in Fig. 1.

## Laboratory assessment

### Blood samples collection

After overnight fast, blood samples from autistics and matched controls were drawn by a qualified lab technician. Blood was taken into three ml blood collection tubes containing EDTA, and samples were immediately centrifuged at 4 °C at 3000 g for 20 min and stored at −80 °C until analysis.

### Biochemical analysis

Chloroform and methanol used for phospholipid extraction, ammonia (NH_3_) and water methanol for the mobile phase were 99% HPLC grade and obtained from Sigma-Aldrich (Taufkirchen, Germany). PC, PS and PE were obtained from Fluka, Sigma-Aldrich (Taufkirchen, Germany).

### Phospholipids assay

#### Phospholipids measurement

Phospholipid separation was performed on a Kaneur Maxi Star HPLC system with four solvent lines, a degasser SEDEX 55 evaporating light detector (SEDEX 55 Lichtstreu detector, S.E.D.E.E., France) which was coupled with Apex M625 software (Autochrom, USA). As the nebulizing gaz, N_2_ was used at a flow rate of 4 l/min, and a nebulizing temperature of 40 °C. The gain was set at 8 and 2.0 bar N_2_.

A 125 × 4.0 mm Si-60 column with 5 μm particle diameter (Lichrosher) was used. The elution program was a linear gradient with 80:19.5:0.5 (V/V) chloroform: methanol: water: ammonia (NH_3_) at 22 min and the column was allowed to equilibrate until the next injection at 27 min. The injection volume was 50 μl. A liquid phase extraction procedure adapted from the method described by Bligh and Dyer [42] was used to extract the serum samples. Briefly, 50 μl of sample was diluted with 750 μl deionized water and mixed well. Then 2 ml of methanol and 1 ml of chloroform were added to the sample and mixed well. Then the mixture was homogenized (Rotary mixture 34,526, Snijders) for 15 min. The mixture was centrifuged for 5 min by 4000 rpm [42].

### Assay of cPLA2

cPLA2 concentration was measured according to the manufacture’s instruction using a competitive enzyme immunoassay technique a product of Amsbio, Blue Gene Company. The detection range of the product was 1.56 ng/ml-100nglml.

### Statistical analysis

SPSS computer program was used. Results were expressed as mean ± SD and all statistical comparisons were made by means of independent *t*-test with *P* ≤0.05 considered as significant. To test the specificity and sensitivity of phospholipids and cPLA2 as markers of autism phenotype, receiver operating characteristics (ROC) analysis was performed. The correlation between the true positive rate (sensitivity) and the false-positive rate (1-specificty) was represented as a curve. The cutoff point was chosen to minimize the sum of false-positive and false-negative test results. The area under the curve (AUC) provides a useful metric to compare different biomarkers. Whereas an AUC value close to 1 indicates an excellent diagnostic and predictive marker, a curve that lies close to the diagonal (AUC = 0.5) has no diagnostic utility. AUC close to 1 is always accompanied by satisfactory values of specificity and sensitivity of the biomarker.

Predictivness curve offer the ability to provide the new risk for an individual based on biomarker test results. It is useful for assessing the fit of the risk model and the classification performance of the biomarker. A horizontal line of the disease prevalence is included as a reference for completely uninformative risk model. Better models will have larger area, below the horizontal disease prevalence line and above predictivness curve, and above the disease prevalence line and below the predictivness curve. In this regard, predictivness curves are the mirror images of ROC curve. Predictiveness diagrams of the measured parameters were drawn in which the x axis represents percentile rank of the biomarker, the y axis represents the probability of identifying the disease, and the horizontal line is the prevalence of the disease using a Biostat 16 computer program. Pearson’s correlations analysis was used to determine the relationships between the parameters and scales.

## Results

**Fig. 2:**
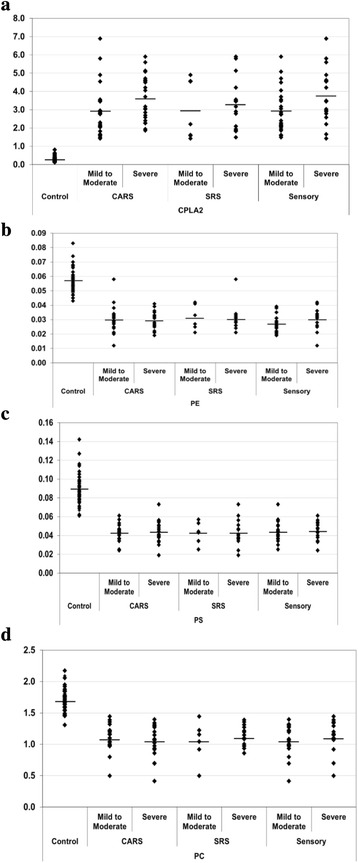
**a**: PE (mmol/L), (**b**) PS (mmol/L), (**c**) PC (mmol/L) (**d**) cPLA2 levels of control and autistic groups. The mean value for each group is designated by a *line*

The significant difference noted in mean plasma concentration of cPLA2 between the autism and control individuals is presented in Table 1 and Fig. 2a. We can easily noticed that autistic patients showed an elevated mean of cPLA2 concentration of 3.242 ng/mL (SD ± 1.345), whereas the control individuals exhibited a much lower mean concentration of only 0.298 ng/mL (SD ± 0.174), *p* < 0.001.

**Table 1:** Levels of phospholipids and cPLA2 in autistic patients compared to control participants

Parameters	Group	N	Mean ± S.D.	*P* value
cPLA2 (ng/ml)	Control	40	0.298 ± 0.174	0.001
	Autistic Patients	48	3.242 ± 1.345	
	Autism (severe in CARS)	24	2.943 ± 1.408	0.083
	Autism (mild to moderate in CARS)	22	3.640 ± 1.242	
	Autism (severe in SRS)	7	2.973 ± 1.608	0.632
	Autism (mild to moderate in SRS)	15	3.304 ± 1.430	
	Autism (mild to moderate in sensory)	25	2.932 ± 1.178	0.050
	Autism (severe in sensory)	18	3.753 ± 1.488	
	Age (less than 7 years)	24	3.234 ± 1.472	0.830
	Age (more than 7)	22	3.322 ± 1.265	
PE (mmole/l)	Control	39	0.057 ± 0.008	0.001
	Autistic Patients	40	0.029 ± 0.008	
	Autism (severe in CARS)	20	0.030 ± 0.009	0.761
	Autism (mild to moderate in CARS)	20	0.029 ± 0.006	
	Autism (severe in SRS)	6	0.031 ± 0.009	0.762
	Autism (mild to moderate in SRS)	14	0.030 ± 0.009	
	Autism (mild to moderate in sensory)	21	0.027 ± 0.006	0.139
	Autism (severe in sensory)	14	0.030 ± 0.008	
	Age (less than 7 years)	20	0.031 ± 0.006	0.319
	Age (more than 7)	18	0.028 ± 0.010	
PS (mmole/l)	Control	39	0.089 ± 0.017	0.001
	Autistic Patients	40	0.043 ± 0.011	
	Autism (mild to moderate in CARS)	20	0.043 ± 0.009	0.605
	Autism (severe in CARS)	20	0.044 ± 0.012	
	Autism (mild to moderate in SRS)	6	0.043 ± 0.012	0.952
	Autism (severe in SRS)	14	0.043 ± 0.014	
	Autism (mild to moderate in sensory)	21	0.044 ± 0.011	0.815
	Autism (severe in sensory)	14	0.045 ± 0.010	
	Age (less than 7 years)	20	0.044 ± 0.012	0.831
	Age (more than 7)	18	99.10 ± 11.86	
PC (mmole/l)	Control	39	1.712 ± 0.184	0.001
	Autistic Patients	40	1.076 ± 0.235	
	Autism (mild to moderate in CARS)	20	1.108 ± 0.216	0.396
	Autism (severe in CARS)	20	1.044 ± 0.253	
	Autism (mild to moderate in SRS)	6	1.045 ± 0.323	0.455
	Autism (severe in SRS)	14	1.128 ± 0.167	
	Autism (mild to moderate in sensory)	21	1.051 ± 0.234	0.394
	Autism (severe in sensory)	14	1.125 ± 0.268	
	Age (less than 7 years)	20	1.039 ± 0.280	0.274
	Age (more than 7)	18	1.126 ± 0.183

In the same Table and Figure (b, c, and d), the mean value of phospholipid levels for individuals with autism and healthy control are also illustrated. The individuals with autism showed a decreased in mean concentrations of 0.029, 0.043 and 1.076 mmole/l for PE, PS and PC compared to control phospholipid of 0.057, 0.089, and 1.712 respectively (Table 1 and Fig. 2).

**Fig. 3:**
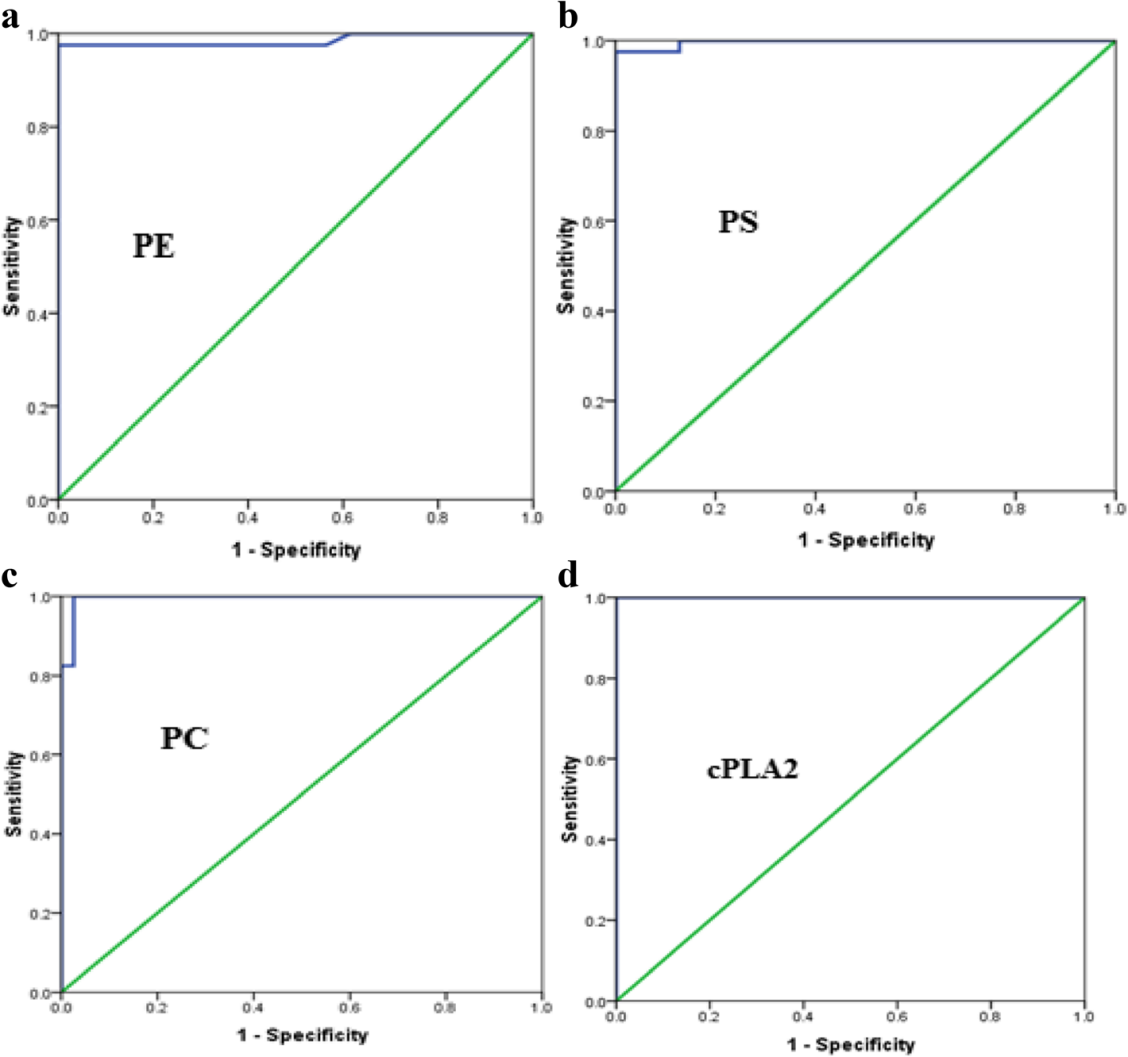
ROC curve (**a**) PE (mmol/L), (**b**) PS (mmol/L), (**c**) PC (mmol/L), (**d**) cPLA2 levels of control and autistic groups

To assess the usefulness of these biomarkers in the diagnosis of autism, ROC analysis was performed. The optimal cut-off points for using PE, PS and PC as biomarkers for autism were 0.043, 0.061 and 1.447mmole/l respectively. These cut-off points were associated with a sensitivity of 97.5, 97.5 and 100% for PE, PS and PC respectively, and a specificity of 100% for all phospholipids have been measured in this study. The optimal cut-off point for using cPLA2as a biomarker for autism was 1.114 ng/ml. This cut-off point was associated with a sensitivity of 100% and a specificity of 100%. (AUC = 1.000) (Fig. 3a and Table 2).

**Table 2:** ROC curves of PE (mmole/l), PS (mmole/l), PC (mmole/l), cPLA2 (ng/ml) in autistic groups

Parameters		Patients with autism	CARS		SRS		SSP	
			Mild to moderate	Severe	Mild to moderate	Severe	Mild to moderate	Severe
PE (mmole/l)	AUC	0.985	0.971	1.000	1.000	0.985	1.000	1.000
	Best cutoff value	0.043	0.043	0.042	0.043	0.039	0.041	0.043
	Sensitivity%	97.5	95	100	92.9	100	100	100
	Specificity%	100	100	100	100	100	100	100
PS (mmole/l)	AUC	0.997	1.000	0.994	1.000	0.991	0.994	1.000
	Best cutoff value	0.061	0.061	0.059	0.059	0.061	0.059	0.061
	Sensitivity%	97.5	100.0	95.0	100.0	92.9	95.2	100.0
	Specificity%	100.0	100.0	100.0	100.0	100.0	100.0	100.0
PC (mmole/l)	AUC	0.996	0.995	0.996	0.996	0.995	0.996	0.993
	Best cutoff value	1.447	1.447	1.422	1.447	1.419	1.422	1.447
	Sensitivity%	100.0	100.0	100.0	100.0	100.0	100.0	100.0
	Specificity%	97.4	97.4	97.4	97.4	97.4	97.4	97.4
cPLA2 (ng/ml)	AUC	1.000	1.000	1.000	1.000	1.000	1.000	1.000
	Best cutoff value	1.114	1.114	1.332	1.114	1.149	1.149	1.114
	Sensitivity%	100.0	100.0	100.0	100.0	100.0	100.0	100.0
	Specificity%	100.0	100.0	100.0	100.0	100.0	100.0	100.0

Demonstrates the ROC analysis data as AUC, cutoff values, specificity, and sensitivity of the measured parameters. All parameters exhibited AUC values close to 1 and satisfactory values of accuracy presented as high specificity and sensitivity

For autistic individuals, the relationship between the levels of phospholipids and severity of autism measured by the CARS, SRS and SSP scores was also evaluated. There were negative correlation between cPLA2 and Phospholipids (i.e., PE, PS, and PC) (*R* = −0.731,-0.757 and −0.741 *p* <0.001).

**Fig. 4:**
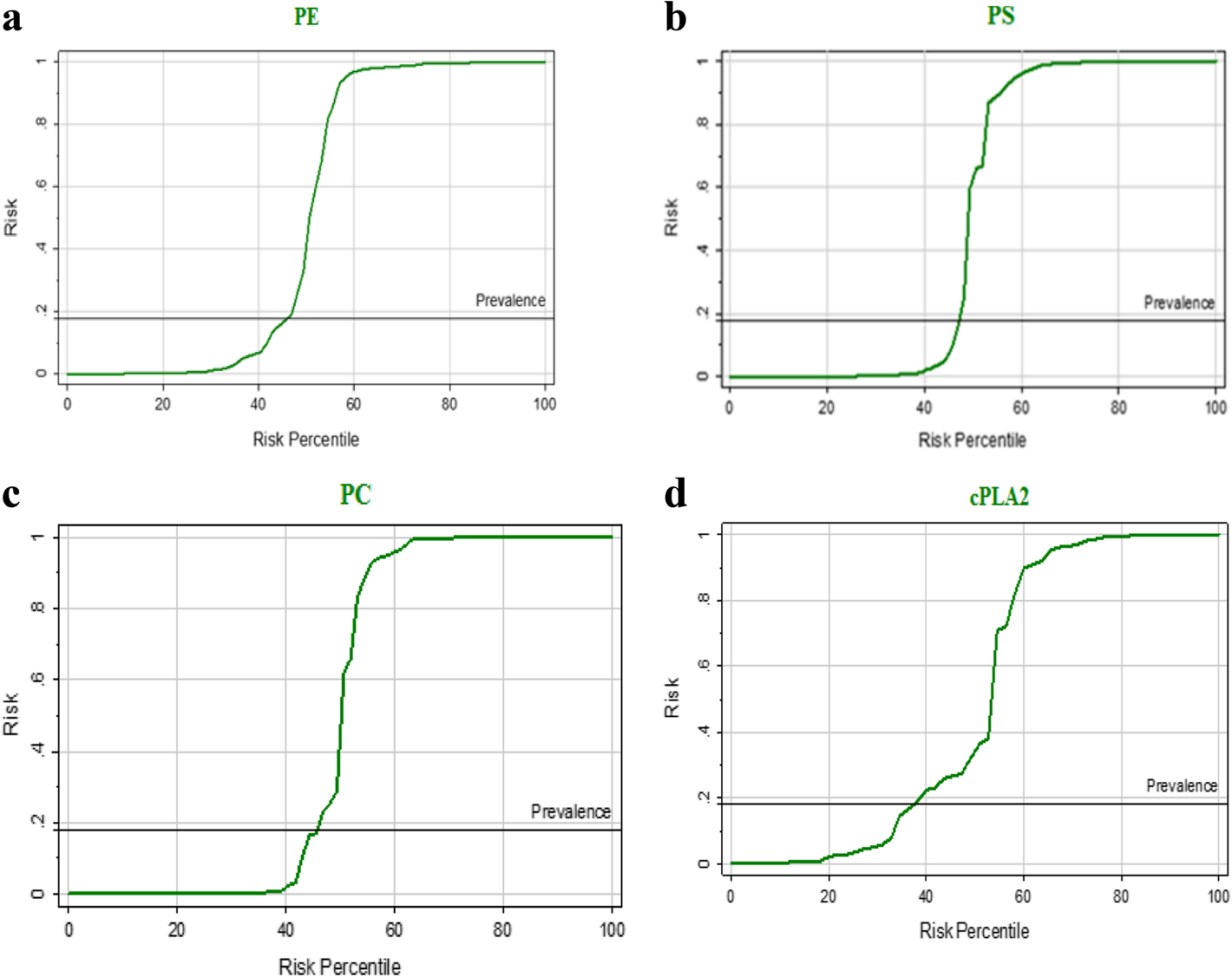
**a**: Predictiveness curve of PE (mmol/L), (**b**) PS (mmol/L), (**c**) PC (mmol/L), (**d**) cPLA2 levels of control and autistic groups

Figure 4 a–d demonstrates the predictiveness curves as an assessment of the performance of phospholipids (i.e., PE, PS and PC) and cPLA2 in autism risk prediction in the Saudi population. The four measured parameters showed adequate predictive power.

## Discussion

This study demonstrates that the levels of phospholipids in children with autism are significantly decreased when compared with typically developing children. Although, variation in phospholipid levels show positive correlation with autism diagnosis but their low level don’t show any association with cognitive and behavioral measures such as stereotypy, hyperactivity, and communication evaluated as CARS and SRS scores. The significant decrease of PE, PS, and PC reported in the present study can find support in many previous studies [36, 43, 44]. Choline plays an important role as a methyl-group donor in the synthesis of phosphotidyl choline that consider as one of essential building blocks for the membrane phospholipid components as well as in the synthesis of the neurotransmitter acetylcholine. Hamlin et al. [45] have been studied the level of choline in plasma of autistic children and healthy controls, they found that autistics had low level of choline compared to controls. Consistent with low choline level, the present study found that PC was significantly lower in the autistic group compared to control.

This study adds further support for a possible role of phospholipid impairment in the neuroinflammation as a pathological mechanism related to autism [46]. This is consistent with reduced levels of PE and enhanced copper-mediated oxidation which was recorded in lymphoblasts from autistic subjects than from control [47, 48]. The significant decrease of PE reported in the present study can be easily related to H_2_O_2_ oxidative stress previously reported in the same Saudi autistic patients, and attributed to the over-expression of SOD together with diminished activity of catalase [49] This can find more support in the work of Glozman et al. [50] which prove that overexpression of Cu/Zn-SOD is correlated to phospholipid depletion.

The anti-inflammatory effect of phospholipids was previously reported by Pandey et al. [51] who found that omega-6 phospholipids, e.g., PC demonstrates anti-inflammatory properties presented as inhibition of tumor necrosis factor (TNF-α) and H_2_O_2_ activated mitogen-activated protein kinase (MAPK) in neuronal SH-SY5Y cell line in addition to the prevention of nuclear factor-kappa B activation by phosphorylation.

Thomas et al. [52] have concluded that after infusion of rodents with propionic acid and butyric acid to be models for ASD, there was alteration in phospholipid profiles. In spite of the non-significant correlation between phospholipids and SSP reported in the present study, change in phospholipid metabolism in autism has been suggested to correlate with language deficits, and this can be observed in autistic children through sensory profile scale that was completed by their parents who always suffer from language difficulty and they need long time to understand what their children want [53].

The reported remarkable increase of cPLA2 concentration in autistic children compared to healthy control (Table 1) can be used to support the previously discussed reduction of phospholipids in autistic plasma. While phospholipids are not correlated with severity of autism, cPLA2 was significantly related to SSP but not CARS, SRS and age. There is evolving evidence for the involvement of cPLA2 in regulating neurite outgrowth and neuronal excitatory functions, both under physiological and pathological conditions. In cultured primary cortical neurons, the stimulation of ionotropic glutamate receptors by *N*-methyl-α-aspartic acid (NMDA) has been shown to activate cPLA2 and AA release [54]. Involvement of cPLA2 in neuronal excitotoxicity has been demonstrated by using neurons from cPLA2 knockout mice, which showed less NMDA-mediated injury as compared to the wild-type controls [55].

Although, there was no correlation between low level of phospholipids and the three measured scales (CARS, SRS, and SSP) as useful measure of cognitive, behavior impairment, and sensory dysfunction in autism [56], have recorded that increased anti-phospholipid antibodies in autism was associated with cognitive and impaired behaviors. The lack of association between cPLA2, CARS and SRS is not in good agreement with certain studies which prove that deregulation of lipid metabolism due to PLA2 over activation is associated with nervous system dysfunction and cognitive impairment [30, 32, 34, 35] which might be attributed to the difference in ethnicity of the present study autistic participants.

A variety of cytokines such as IL-1, TNF-α and IFNγ have been shown to induce activation and increase synthesis of cPLA2 in diverse cell models [57]. In different studies on autistic patients, there were elevated level of those cytokines in autistic children compared to healthy controls which might explain the elevated level of cPLA2 in autistic children compared to control, reported in the present study [58]. In addition, increasing cytokine levels were associated with more impaired aberrant behaviors [59], and this could support the obtained correlation between elevated cPLA2 activity and SSP as measure of sensory processing dysfunction. It is well documented that cPLA2 is involved in injury of primary sensory neurons and pain behavior in the peripheral nervous system and that its inhibition decreases the levels of injurious lipid mediators, reduce pain [60, 61]. Again this can support the obtained correlation between cPLA2 and SSP (Table 1).

Oxidant compounds such as H_2_O_2_ also led to activation of cPLA2, which in turn alters membrane molecular order and cytoskeletal arrangements. H_2_O_2_ causes astrocyte membranes to become more gel-like and induces actin polymerization and, subsequently, enhances formation of cytonemes and cell-to-cell connections, which can affect glutamate transporter and thus induce glutamate excitotoxity [62]. Zhu et al. [63] suggested that oxidative stress may have an important impact on astrocyte membranes, signaling pathways and cytoskeletal arrangements. It is worth noting that under stimulated conditions, AA released by cPLA2 activity is a good target for lipid peroxidation, as lipid peroxides contribute to the increased oxidative pool in the cells [64]. These studies can be supported by Al-Gadani et al. [50] who suggested that autistic children are under H_2_O_2_ stress due to GSH depletion.

Collectively, the previous mentioned studies give strong evidence for the role of cPLA2 in oxidative and inflammatory signaling pathways and provide evidence for its link in the pathogenesis of ASD, and also activation of cytosolic PLA2 (cPLA2) has been shown to produce lipoxidative toxicity, leading to inflammation and pain.

Administration of PS containing Omega3 long-chain polyunsaturated fatty acids to children with attention-deficit/hyperactivity disorder (ADHD) symptoms have been shown to reduce hyperactivity, attention deficits and behavior dysregulations as main characteristics of autism phenotype. Based on this, we can suggest that in spite of the absence of correlation between PS depletion and severity of autism, in the present study, PS supplementation can be used as treatment strategy [65].

The interesting positive correlation between SSP as useful scale of sensory abnormalities and age can be related to the remarkable improvement of autistic patients near adulthood (Table 3). This suggestion can find support in two previous studies that proved the improvement of majority of autistic patient’s phenotype during the transition to adulthood [66, 67].

**Table 3:** Pearson’s correlations between age, CARS, SRS, SSP, and the four measured parameters

Parameters	R (Person Correlation)	Sig.	
SSP ~ Age	0.296^*^	0.043	P^a^
SSP ~ cPLA2	−0.319^*^	0.037	N^b^
cPLA2 ~ PE	−0.731^**^	0.001	N^b^
cPLA2 ~ PS	−0.757^**^	0.001	N^b^
cPLA2 ~ PC	−0.741^**^	0.001	N^b^
PE ~ PS	0.778^**^	0.001	P^a^
PE ~ PC	0.692^**^	0.001	P^a^
PS ~ PC	0.679^**^	0.001	P^a^

^**^ Correlation is significant at *P* < 0.01 level.,^*^Correlation is significant at *P* < 0.05 level

^a^Positive Correlation., ^b^Negative Correlation

The predictiveness curves of the four measured parameters (Fig. 4a–d), varies significantly from the baseline risk depending on whether PE, PS, PC, and cPLA2 concentrations were low or very high. So, these parameters can be used as predictive biomarkers in autism field. This is supported by the high sensitivity and specificity recorded through ROC analysis (Table 2).

## Conclusion

**Fig. 5:**
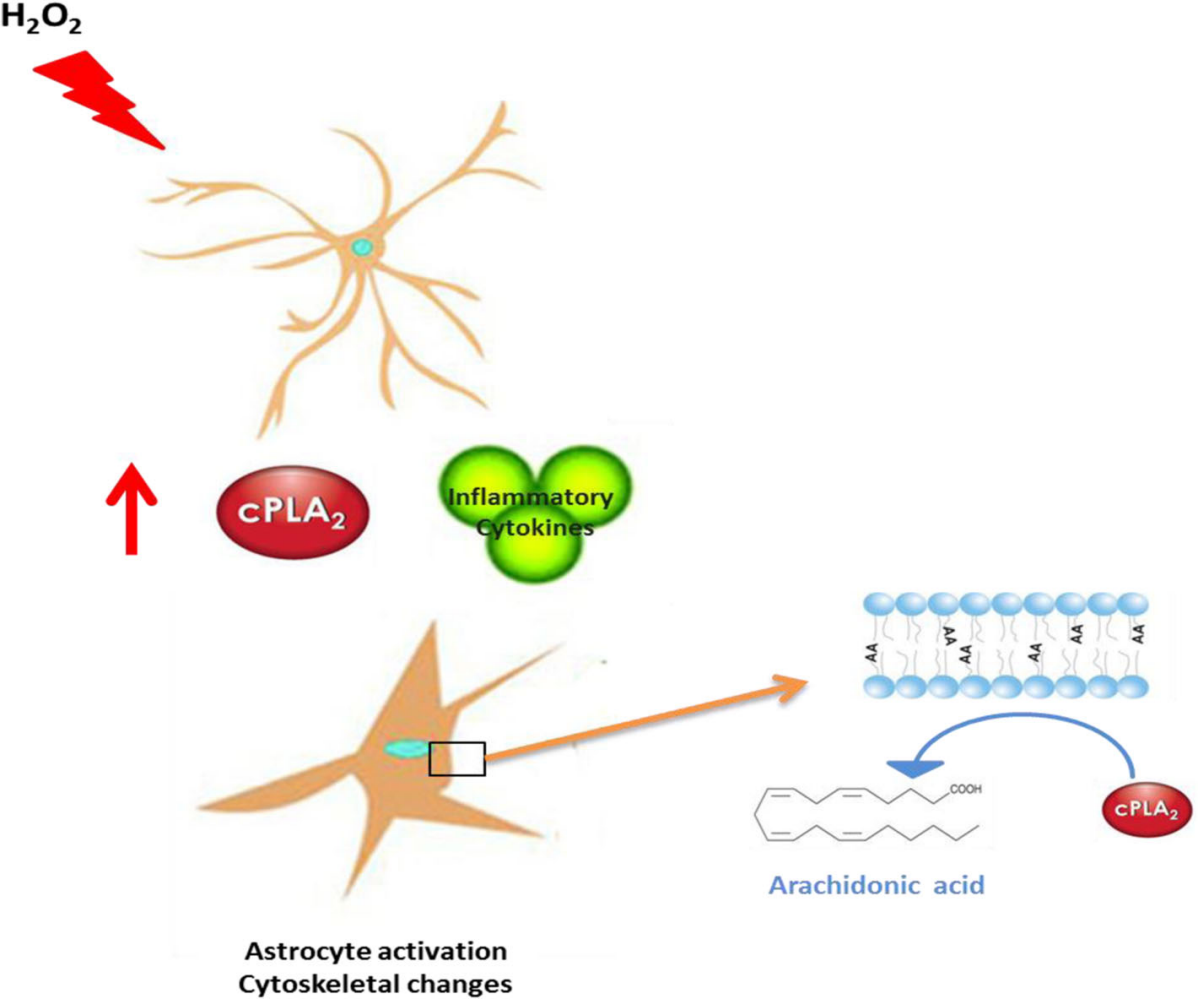
The relationship between impaired phospholipid through the activation of cPLA2, oxidative stress, and neuroinflammation

In the present study, the decreased in phospholipid levels together with increased cPLA2 in children with autism and the correlation between cPLA2 levels and sensory abnormalities offer a potential new target for understanding the mechanisms involved in the pathogenicity of autism. The explained relationship between impaired phospholipid through the activation of cPLA2, oxidative stress, and neuroinflammation can be illustrated in Fig. 5.

## Abbreviations

AA: Arachidonic acid
ADI-R: Autism diagnostic interview-revised
ADOS: Autism diagnostic observation schedule
CARS: Childhood autism rating scale
cPLA2: Cytosolic Phospholipase A2
GSH: Glutathione
IFNγ: Interferon gamma
IL-6: Interleukin-6
NMDA: N-methyl-D-aspartate
PC: Phosphatidyl choline
PE: Phosphatidyl ethanolamine
PS: Phosphatidyl serine
PUFAs: Polyunsaturated fatty acids
ROC-curve: Receiver operating characteristics curve
ROS: Reactive oxygen species
SOD: Superoxide dismutase
SRS: Social responsiveness scale
SSP: Short sensory profile
TNF-α: Tumor necrosis factor alpha

## References

[1] Gordan J. One in Every 50 Children Has Autism. UCLA Medical School CDC; 2013. http://www.huffingtonpost.com/jay-gordon/autismrates_b_2921256.html.

[2] Ozonoff S, Iosif A-M, Baguio F, Cook IC, Hill MM, Hutman T, et al. A prospective study of the emergence of early behavioral signs of autism. J Am Acad Child Adolesc Psychiatry. 2010;49:256–66. e252. PMC2923050 20410715

[3] Klintwall L, Holm A, Eriksson M, Carlsson LH, Olsson MB, Hedvall O (2011). Sensory abnormalities in autism: a brief report. Res Dev Disabil.

[4] King CR (2011). A novel embryological theory of autism causation involving endogenous biochemicals capable of initiating cellular gene transcription: a possible link between twelve autism risk factors and the autism ‚Äòepidemic‚Äô. Med Hypotheses.

[5] Sung YJ, Dawson G, Munson J, Estes A, Schellenberg GD, Wijsman EM (2005). Genetic investigation of quantitative traits related to autism: use of multivariate polygenic models with ascertainment adjustment. Am J Hum Genet.

[6] Lombardo MV, Barnes JL, Wheelwright SJ, Baron-Cohen S (2007). Self-referential cognition and empathy in autism. PLoS One.

[7] Itzchak EB, Lahat E, Burgin R, Zachor AD (2008). Cognitive, behavior and intervention outcome in young children with autism. Res Dev Disabil.

[8] Grandin T. Thinking in pictures, expanded edition: My life with autism. Vintage; 2009.

[9] Blackman L. Lucy’s story: Autism and other adventures. England: Jessica Kingsley Publishers; 2001.

[10] Rogers SJ, Hepburn S, Wehner E (2003). Parent reports of sensory symptoms in toddlers with autism and those with other developmental disorders. J Autism Dev Disord.

[11] Tomchek SD, Dunn W (2007). Sensory processing in children with and without autism: a comparative study using the short sensory profile. Am J Occup Ther.

[12] Pardo CA, Vargas DL, Zimmerman AW (2005). Immunity, neuroglia and neuroinflammation in autism. Int Rev Psychiatry.

[13] Morgan JT, Chana G, Pardo CA, Achim C, Semendeferi K, Buckwalter J (2010). Microglial activation and increased microglial density observed in the dorsolateral prefrontal cortex in autism. Biol Psychiatry.

[14] Theoharides TC, Doyle R (2008). Autism, gut-blood-brain barrier, and mast cells. J Clin Psychopharmacol.

[15] Skaper SD, Giusti P, Facci L (2012). Microglia and mast cells: two tracks on the road to neuroinflammation. FASEB J.

[16] Vargas DL, Nascimbene C, Krishnan C, Zimmerman AW, Pardo CA (2005). Neuroglial activation and neuroinflammation in the brain of patients with autism. Ann Neurol.

[17] Zimmerman AW, Jyonouchi H, Comi AM, Connors SL, Milstien S, Varsou A (2005). Cerebrospinal fluid and serum markers of inflammation in autism. Pediatr Neurol.

[18] James SJ, Cutler P, Melnyk S, Jernigan S, Janak L, Gaylor DW (2004). Metabolic biomarkers of increased oxidative stress and impaired methylation capacity in children with autism. Am J Clin Nutr.

[19] Main PA, Angley MT, O’Doherty CE, Thomas P, Fenech M (2012). The potential role of the antioxidant and detoxification properties of glutathione in autism spectrum disorders: a systematic review and meta-analysis. Nutr Metab.

[20] Rossignol DA, Frye RE (2014). Evidence linking oxidative stress, mitochondrial dysfunction, and inflammation in the brain of individuals with autism. Front Physiol.

[21] Chiurchiu V, Maccarrone M (2011). Chronic inflammatory disorders and their redox control: from molecular mechanisms to therapeutic opportunities. Antioxid Redox Signal.

[22] Farfara D, Lifshitz V, Frenkel D (2008). Neuroprotective and neurotoxic properties of glial cells in the pathogenesis of Alzheimer’s disease. J Cell Mol Med.

[23] Fuller S, Steele M, Munch G (2010). Activated astroglia during chronic inflammation in Alzheimer’s disease‚Äîdo they neglect their neurosupportive roles?. Mutat Res.

[24] Moreira PI, Smith MA, Zhu X, Nunomura A, Castellani RJ, Perry G (2005). Oxidative stress and neurodegeneration. Ann N Y Acad Sci.

[25] Melo A, Monteiro L, Lima RM, de Oliveira DgM, de Cerqueira MD, El-Bach RS. Oxidative stress in neurodegenerative diseases: mechanisms and therapeutic perspectives. Oxid Med Cell Longev. 2011;2011:1–14. 10.1155/2011/467180 PMC3236428 22191013

[26] Streit WJ, Mrak RE, Griffin WST (2004). Microglia and neuroinflammation: a pathological perspective. J Neuroinflammation.

[27] Horrobin D, Bennett C. Phospholipid metabolism and the pathophysiology of psychiatric and neurological disorders. Phospholipid Spectr Dis Psychiatry Neurol. 2003:3–47.

[28] Maxfield FR, Tabas I (2005). Role of cholesterol and lipid organization in disease. Nature.

[29] Richardson A (2003). Phospholipid spectrum disorders in psychiatry and neurology.

[30] Morales I, Guzm√°n-Mart√ ≠ nez L, Cerda-Troncoso Cb, Far√ ≠ as GA, Maccioni RB (2015) Neuroinflammation in the pathogenesis of Alzheimer‚Äôs disease. A rational framework for the search of novel therapeutic approaches. 2015: Which new directions for Alzheimer’s disease? 10.3389/fncel.2014.00112 PMC4001039 24795567

[31] Aoyama K, Watabe M, Nakaki T (2008). Regulation of neuronal glutathione synthesis. J Pharmacol Sci.

[32] Bell J, MacKinlay E, Dick J, MacDonald D, Boyle R, Glen A (2004). Essential fatty acids and phospholipase A 2 in autistic spectrum disorders. Prostaglandins Leukot Essent Fat Acids.

[33] Pastural A, Ritchie S, Lu Y, Jin W, Kavianpour A, Su-Myat KK (2009). Novel plasma phospholipid biomarkers of autism: mitochondrial dysfunction as a putative causative mechanism. Prostaglandins Leukot Essent Fat Acids.

[34] Adibhatla RM, Hatcher J (2008). Phospholipase A2, reactive oxygen species, and lipid peroxidation in CNS pathologies. BMB Rep.

[35] Sanchez-Mejia RO, Mucke L (2010). Phospholipase A 2 and arachidonic acid in Alzheimer’s disease. Biochim Biophys Acta.

[36] El-Ansary AK, Bacha AGB, Al-Ayahdi LY (2011). Plasma fatty acids as diagnostic markers in autistic patients from Saudi Arabia. Lipids Health Dis.

[37] Tostes M, Teixeira H, Gattaz W, Brandão M, Raposo N (2012). Altered neurotrophin, neuropeptide, cytokines and nitric oxide levels in autism. Pharmacopsychiatry.

[38] Hermann PM, Park D, Beaulieu E, Wildering WC (2013). Evidence for inflammation-mediated memory dysfunction in gastropods: putative PLA 2 and COX inhibitors abolish long-term memory failure induced by systemic immune challenges. BMC Neurosci.

[39] Mick KA. Diagnosing autism: Comparison of the childhood autism rating scale (CARS) and the autism diagnostic observation schedule (ADOS). Kansas: Wichita State University; 2005.

[40] Constantino JN, Davis SA, Todd RD, Schindler MK, Gross MM, Brophy SL (2003). Validation of a brief quantitative measure of autistic traits: comparison of the social responsiveness scale with the autism diagnostic interview-revised. J Autism Dev Disord.

[41] Dunn W (2001). The sensations of everyday life: empirical, theoretical, and pragmatic considerations. Am J Occup Ther.

[42] Bligh EG, Dyer WJ (1959). A rapid method of total lipid extraction and purification. Can J Biochem Physiol.

[43] Chauhan A, Chauhan V, Brown WT, Cohen I (2004). Oxidative stress in autism: Increased lipid peroxidation and reduced serum levels of ceruloplasmin and transferrin - the antioxidant proteins. Life Sci.

[44] El-Ansary AK, Bacha AGB, Al-Ayadhi LY (2011). Impaired plasma phospholipids and relative amounts of essential polyunsaturated fatty acids in autistic patients from Saudi Arabia. Lipids Health Dis.

[45] Hamlin JC, Pauly M, Melnyk S, Pavliv O, Starrett W, Crook TA (2013). Dietary intake and plasma levels of choline and betaine in children with autism spectrum disorders. Autism Res Treat.

[46] El-Ansary A, Al-Ayadhi L (2012). Neuroinflammation in autism spectrum disorders. J Neuroinflammation.

[47] Chauhan A, Sheikh AM, Chauhan V (2008). Increased copper-mediated oxidation of membrane phosphatidylethanolamine in autism. Am J Biochem Biotechnol.

[48] Bell JG, Sargent JR, Tocher DR, Dick JR (2000). Red blood cell fatty acid compositions in a patient with autistic spectrum disorder: a characteristic abnormality in neurodevelopmental disorders?. Prostaglandins Leukot Essent Fatty Acids.

[49] Al-Gadani Y, El-Ansary A, Attas O, Al-Ayadhi L (2009). Metabolic biomarkers related to oxidative stress and antioxidant status in Saudi autistic children. Clin Biochem.

[50] Glozman S, Cerruti-Harris C, Groner Y, Yavin E (2000). Docosahexaenoic acid-deficient phosphatidyl serine and high Œ ± −tocopherol in a fetal mouse brain over-expressing Cu/Zn-superoxide dismutase. Biochim Biophys Acta.

[51] Pandey N, Sultan K, Twomey E, Sparks D (2009). Phospholipids block nuclear factor-kappa B and tau phosphorylation and inhibit amyloid-beta secretion in human neuroblastoma cells. Neuroscience.

[52] Thomas RH, Foley KA, Mepham JR, Tichenoff LJ, Possmayer F, MacFabe DF (2010). Altered brain phospholipid and acylcarnitine profiles in propionic acid infused rodents: further development of a potential model of autism spectrum disorders. J Neurochem.

[53] Minshew NJ, Goldstein G, Dombrowski SM, Panchalingam K, Pettegrew JW (1993). A preliminary 31P MRS study of autism: evidence for undersynthesis and increased degradation of brain membranes. Biol Psychiatry.

[54] Shelat PB, Chalimoniuk M, Wang JÄ, Strosznajder JB, Lee JC, Sun AY (2008). Amyloid beta peptide and NMDA induce ROS from NADPH oxidase and AA release from cytosolic phospholipase A2 in cortical neurons. J Neurochem.

[55] Shen Y, Kishimoto K, Linden DJ, Sapirstein A (2007). Cytosolic phospholipase A2 alpha mediates electrophysiologic responses of hippocampal pyramidal neurons to neurotoxic NMDA treatment. Proc Natl Acad Sci.

[56] Careaga M, Hansen RL, Hertz-Piccotto I, Van de Water J, Ashwood P (2013). Increased anti-phospholipid antibodies in autism spectrum disorders. Mediators Inflamm.

[57] Goines PE, Ashwood P (2013). Cytokine dysregulation in autism spectrum disorders (ASD): possible role of the environment. Neurotoxicol Teratol.

[58] Enstrom AM, Onore CE, Van de Water JA, Ashwood P (2010). Differential monocyte responses to TLR ligands in children with autism spectrum disorders. Brain Behav Immun.

[59] Ashwood P, Krakowiak P, Hertz-Picciotto I, Hansen R, Pessah I, Van de Water J (2011). Elevated plasma cytokines in autism spectrum disorders provide evidence of immune dysfunction and are associated with impaired behavioral outcome. Brain Behav Immun.

[60] Tsuda M, Hasegawa S, Inoue K (2007). P2X receptors‚Äêmediated cytosolic phospholipase A2 activation in primary afferent sensory neurons contributes to neuropathic pain. J Neurochem.

[61] Khan M, Shunmugavel A, Dhammu TS, Matsuda F, Singh AK, Singh I (2015). Oral administration of cytosolic PLA2 inhibitor arachidonyl trifluoromethyl ketone ameliorates cauda equina compression injury in rats. J Neuroinflammation.

[62] Sun GY, Shelat PB, Jensen MB, He Y, Sun AY, Simonyi A (2010). Phospholipases A2 and inflammatory responses in the central nervous system. Neruomol Med.

[63] Zhu D, Tan KS, Zhang X, Sun AY, Sun GY, Lee JC-M (2005). Hydrogen peroxide alters membrane and cytoskeleton properties and increases intercellular connections in astrocytes. J Cell Sci.

[64] Nanda B, Nataraju A, Rajesh R, Rangappa K, Shekar M, Vishwanath B (2007). PLA2 mediated arachidonate free radicals: PLA2 inhibition and neutralization of free radicals by anti-oxidants-a new role as anti-inflammatory molecule. Curr Top Med Chem.

[65] Manor I, Magen A, Keidar D, Rosen S, Tasker H, Cohen T (2012). The effect of phosphatidylserine containing Omega3 fatty-acids on attention-deficit hyperactivity disorder symptoms in children: a double-blind placebo-controlled trial, followed by an open-label extension. Eur Psychiatry.

[66] Taylor JL, Seltzer MM (2010). Changes in the autism behavioral phenotype during the transition to adulthood. J Autism Dev Disord.

[67] Shattuck PT, Seltzer MM, Greenberg JS, Orsmond GI, Bolt D, Kring S (2007). Change in autism symptoms and maladaptive behaviors in adolescents and adults with an autism spectrum disorder. J Autism Dev Disord.

